# Prevalence of sustainability claims on packaged food

**DOI:** 10.1017/S1368980026102511

**Published:** 2026-04-28

**Authors:** Mariel Keaney, Eden M. Barrett, Mike Rayner, Simone Pettigrew, Alexandra Jones

**Affiliations:** 1 The George Institute for Global Health, University of New South Wales, Sydney, Australia; 2 School of Health Sciences, Faculty of Medicine and Health, University of New South Wales, Sydney, Australia; 3 University of Oxford, UK

**Keywords:** Food environments, Food labelling, Food sustainability, Eco-labelling, Sustainability claims

## Abstract

**Objective:**

Global food systems have a key influence on both health and sustainability. Dietary shifts that promote health and sustainability are regarded as a critical synergistic pathway for advancing human and planetary health. In response, consumer interest in the sustainability of food systems has prompted the use of claims highlighting positive sustainability attributes displayed on-pack. This study assessed the prevalence and types of sustainability claims displayed on packaged food in Australia.

**Design:**

Claims data on packaged food were collected at five supermarkets in Sydney in 2024. We determined the prevalence of sustainability claims overall, by claim category and format (text or logo).

**Setting:**

Australia.

**Participants:**

None.

**Results:**

Over a third of products displayed at least one sustainability claim, the most prevalent being ‘natural’ and ‘vegan’. Text claims were far more prevalent than logos (84 % *v* 16 % of total claims identified on pack, respectively).

**Conclusions:**

Frequent use of broad and unspecific sustainability claims raises challenges for substantiation, and there is a risk that consumers are being misled. Our findings support the need for stronger regulation, including criteria for terms used in claims, to improve consumer trust and comparability across products.

Food systems are becoming increasingly globalised and complex, which brings a wide range of negative health, environmental and social ramifications. Health consequences include the ‘triple burden’ of malnutrition observed worldwide, where hunger, malnutrition and obesity occur simultaneously^(1)^. Food production is among the largest drivers of environmental change globally^(2)^, with negative impacts on climate change, biodiversity loss and pollution. It is estimated that between 21 % and 37 % of global greenhouse gas emissions are attributed to the food system^(2,3)^. Inhumane treatment of animals can exacerbate environmental degradation, such as water pollution and greenhouse gas emissions, and increase their susceptibility to disease^(4)^. Further, power imbalances in the food system have led to social ramifications for farmers and workers. These include imbalanced wealth distribution among farmers, such as government subsidies biased towards large operations instead of small-scale farming, and child labour^(5,6)^.

These wide-ranging impacts are commonly conceptualised as falling within different ‘sustainability domains’. In this paper, we refer to the domains of environmental sustainability, animal welfare and social sustainability^(7)^. Environmental sustainability considers the planetary boundaries of climate change, nitrogen and phosphorous cycling, water use, biodiversity or land use change^(2)^. Animal welfare is concerned with the treatment of animals, including living conditions, nutrition and mental well-being^(4)^. Social sustainability involves the stakeholders throughout the food supply chain, including farmers and their communities so that they are treated fairly^(6)^.

Synergies exist between foods that support both human health and the sustainability domains. For example, there is evidence to suggest healthier diets are correlated with low environmental footprints^(8)^. The negative ramifications of food systems have led to growing consumer interest in food choices that are both healthy and sustainable^(9)^. Similar to nutrition labelling, sustainability information displayed on products has the potential to guide consumer purchase behaviour towards more sustainable choices and incentivise the food industry to improve the sustainability of their practices^(10,11)^.

In light of consumer interest in sustainability information, various sustainability labels on food products have emerged that may be self-declared by manufacturers, required by governments and/or endorsed by third parties^(7,12,13)^. Such labels can be presented as text claims (e.g. ‘vegan’ or ‘plant based’) or graphical logos (e.g. ‘Certified Organic’). For sustainability labelling to be useful in transitioning to sustainable food environments, the information and claims displayed must be accurate and meaningful. As defined by the international food standards agency, the Codex Alimentarius Commission (Codex) and the Australian Competition and Consumer Commission (ACCC), accurate labels are representative of a product’s attributes without exaggerating their benefits^(10,11,14)^. In order for sustainability labels be meaningful, consumers must be able to understand and interpret them. However, the development of accurate and meaningful sustainability labels is challenging because regulation in this area is in its infancy compared with nutrition labelling. At a global level, Codex provides accepted global norms that guide national policymakers in regulating health and nutrition claims to ensure they are clear and not misleading^(15,16)^. While Codex has considered developing equivalent international standards for sustainability labels, this work is at a very early stage^(11)^.

Monitoring the use of sustainability labels is necessary to understand the policy priorities to genuinely steer consumers towards more sustainable food choices^(14)^. Due to the diversity of labels, effective monitoring supported by categorising the different types of sustainability labels identified. To address this, a recent taxonomy has been developed to categorise sustainability labels, based on a similar approach for health and nutrition claims^(14,17)^. Under this taxonomy, labels are distinguished as either ‘information’ or ‘claims’.

Information labels are typically required by legislation or are government endorsed and inform the consumer about the properties of the food in a neutral fashion or warn them of negative characteristics^(17)^. Information labelling is commonly used in nutrition communication (e.g. ingredient declarations, nutritional information panels), but globally there are few examples of informational labelling that is specifically related to sustainability^(7,17)^. The French Eco-score, developed with governmental involvement, is one example that generates an overarching sustainability rating from A to E based on multiple sustainability indicators, with an A rating denoting more sustainable products, and an E rating for less sustainable products^(12)^.

By contrast, sustainability claims are voluntarily made to highlight positive attributes only, such as ‘carbon friendly’ or ‘NoCO2 Certified’^(17)^. Criticisms of the substantial growth in the number of sustainability claims include consumers becoming confused when attempting to differentiate between a plethora of claims, and being misled where products’ sustainability benefits are overstated (known as ‘greenwashing’)^(18,19)^. Further, claims can be considered greenwashing if they are too broad and ambiguous, or omit necessary information to evaluate their validity^(20)^.

Existing studies demonstrate a growing consumer preference for products displaying sustainability labels, particularly among those already concerned about the environment^(9,21)^. Although sustainability labels are known to steer consumers towards more sustainable products, research to date has predominantly focussed on consumer preference for individual labels^(9,21)^. There has been little systematic assessment of their prevalence and no application of systematic frameworks to categorise the diverse claims in use. Codex’s work to date stocktaking existing labels demonstrates the wide variety of sustainability logos that exist globally, but does not assess how frequently these are displayed in practice^(7)^. The few prevalence studies conducted to date are limited in product scope (e.g. focusing on specific product categories or only new products) or limited to a selection of sustainability labels (e.g. only look at the uptake of a particular logo)^(22,23)^.

In light of this, the aim of this study was to pilot a systematic approach to monitor the prevalence of sustainability claims by (i) assessing the frequency and variety of sustainability claims identified in a representative sample of packaged foods available for sale in major Australian supermarkets and (ii) analysing these claims using an adapted taxonomy for categorising sustainability claims that includes consideration of sustainability domains, claim types and text or logo format.

## Methods

### Data source

The FoodSwitch database, developed by The George Institute for Global Health, contains annually updated product-level information on packaged foods and beverages available for sale in Australia, including food composition and labelling information^(24)^. Our analysis used the 2024 Australian FoodSwitch Monitoring Dataset. Data collection occurred between March and June 2024 at five supermarkets located in metropolitan Sydney (Coles, Woolworths, IGA, Aldi and Harris Farm). Photographs of products and their labels were taken in-store and then systematically recorded as per previously developed protocol for the FoodSwitch database^(24)^.

Identified products were assigned to a major food and beverage category based on the Global Food Monitoring categorisation system^(25)^. We excluded non-food categories (i.e. alcoholic beverages and vitamins and supplements), leaving 27 738 products covering ffiteen major food categories for analysis.

### Categorisation of sustainability claims

Claims were deemed to be sustainability claims if they were relevant to at least one sustainability theme, or ‘domain’: animal welfare, environmental, social or generic. For the purposes of this study, sustainability claims included text claims and graphical logos displaying positive sustainability attributes on any surface of a product’s packaging, including within the product name and brand. We determined prevalence by identifying the number of different sustainability claims displayed on a product’s packaging. For example, a product displaying ‘carbon friendly’, ‘natural’ and ‘vegan’ was considered to have three different claims on pack. Where a claim was repeated on a product, for example, where ‘natural’ appeared in both the brand name and elsewhere, we counted that claim only once. The number of different sustainability claims on each product package was aggregated across the overall sample to arrive to the total prevalence of claims.

#### Application of an international taxonomy of sustainability labels

We adapted a taxonomy developed for the International Network for Food and Obesity/non-communicable diseases Research, Monitoring and Action Support (INFORMAS) to categorise sustainability labels identified on food packages in Australia^(17)^. While the taxonomy considers both claims and information labels, the present study included only claims given that Australia currently has no regulated requirements for sustainability information, therefore by definition there are no information labels in the market.

The INFORMAS taxonomy categorises sustainability claims by distinct aspects of sustainability (‘sustainability domains’). The sustainability domains adopted from the INFORMAS taxonomy are animal welfare, environmental and social. Some sustainability claims that could arguably also be health related were included in our analysis. For example, ‘organic’ and ‘vegan’ claims can relate to environmental sustainability and animal welfare, but could also be considered to relate to health (see online supplementary material, Supplemental Table 1 for included sustainability claims that could also have a health inference).

Where a domain could not easily be ascertained (e.g. the claim ‘sustainable’, that could refer to all domains, or only some of the domains), sustainability claims were categorised into an additional ‘generic’ sustainability domain. Where a claim referred to more than one sustainability domain, it was classified as ‘multi-domain’. For example, the Fairtrade Certification covers multiple criteria across social and environmental sustainability domains, including child labour, biodiversity and prohibited use of GM organisms^(26)^. For the purposes of this study, we considered ‘vegan’ claims to be multi-domain given they incorporate both animal welfare and environmental considerations. Overall, five sustainability domains were included. Table 1 contains examples of claims in each domain (see online supplementary material, Supplemental Table 1 for the full list of identified claims and their allocated domain).

**Table 1:** Classification of sustainability claims and examples, adapted from the INFORMAS taxonomy

Sustainability domain	Claim type			
	Ingredient	Production^*^	Whole life cycle^†^	Unspecified
Animal welfare		Free range RSPCA Approved		
Environment		Free from chemicals Marine Stewardship Council Certified	Carbon Neutral Certified	Eco friendly Natural
Social		Fairtrade Farmer friendly		
Generic	100 % palm oil free	Responsibly fished		Sustainable palm oil
Multi-domain	No animal content Vegan	GMO free Organic		

* Referred to as ‘Component claims’ according to the INFORMAS taxonomy.

† Referred to as ‘Impact claims’ according to the INFORMAS taxonomy.

Under these domains, sustainability claims were further categorised by type, depending on the element of a product’s sustainability that the claim referred to (see Table 1 for examples). Types of sustainability claims include those relating to ingredients (‘ingredient claims’), production processes (‘production claims’) or a comprehensive whole life cycle assessment of the product (‘whole life cycle claims’). Claims were classified as ‘ingredient claims’ if they referred to substances included in or absent from the final product (e.g. ‘plant based’ and ‘100 % palm oil free’). Claims were classified as ‘production claims’ if they referred to production processes and activities during the farming stage (e.g. sustainable farming practices, minimising use of pesticides and practices that limit emissions). Claims classified as ‘whole life cycle claims’ were based on a product’s life cycle impact. Life cycle approaches measure the impact of products throughout their life cycles, considering raw materials, production, transport, retail and consumption (e.g. ‘Carbon Neutral Certified’)^(27)^. Claims that could not be categorised into a specific type were categorised as ‘unspecified’, such as ‘natural’ claims that can refer to a product’s ingredients and/or production process. For the purposes of this research, only claims referring to the product or its production were included, therefore claims regarding a product’s packaging (e.g. recycling) were excluded.

#### Analysis of text or logo format

In addition to the existing categorisations included in the INFORMAS taxonomy, we analysed claims based on whether they were presented in text or logo format. Text claims are terms or phrases featured on product packaging and are typically self-declared by the manufacturer without independent verification. Logos are graphics and/or symbols, such as the green frog trademark for Rainforest Alliance, that are usually certified by independent third-party organisations who verify that the product meets a specific standard. Being a pictorial format, logos can assist consumers in easily identifying products that align with their sustainability values. Claims relating to the same issue can sometimes be presented in both formats, for example, a text claim of ‘vegan’ and a ‘Vegan Australia Certified’ logo.

One coder assessed each different claim in the dataset and classified claims by sustainability domain, claim type and format. These initial classifications were then reviewed and refined by the broader authorship team to reach final agreement. See online supplementary material, Supplemental Table 1 for the full list of identified claims and their allocated sustainability domain, claim type and explanations for their classification. See online supplementary material, Supplemental Table 2 for the format for each identified claim.

#### Statistical analysis

We measured prevalence first by calculating the number and percentage of products displaying at least one sustainability claim from the total products in the sample. We also identified the number of different claims on each product package aggregated across the overall sample. To understand the variety of sustainability claims used, we calculated the number of claims identified at least once across all products in the sample. Analyses were conducted overall and by major food category. Given potentially large difference in sustainability impacts within the category of ‘meat and meat alternatives’, we conducted additional sub-category analysis for animal-based meat products (‘meat’) and plant-based meat alternatives (‘meat alternatives’).

Using the adapted INFORMAS typology, each sustainability claim was assigned to one sustainability domain and type of claim. We conducted the same overall analysis described above separately for each sustainability domain and claim type and for each text or logo format. We also quantified the proportion of claims assigned to each sustainability domain and claim type to assess overall distribution. Analyses were conducted in Stata/BE version 18·0.

## Results

### Overall prevalence

Overall, 10 781 of 27 738 (39 %) products made at least one sustainability claim on pack (see Table 2), and there were a total of 17 423 different instances of sustainability claims identified on packs. The sample contained a variety of sixty-nine sustainability claims. ‘Natural’ was the most prevalent claim (24 % of claims), followed by ‘vegan’ (21 %). These two claims represented 15 % and 13 % of claims on all products, respectively (see online supplementary material, Supplemental Table 2 for the prevalence of each claim identified). Both claims were consistently prominent at the major food category level. The ‘natural’ claim was amongst the most prevalent in all product categories. The ‘vegan’ claim featured prominently in twelve of fifteen product categories (see Table 2). Although a large proportion of meat alternatives featured the ‘vegan’ claim (63 %, data not shown), this product category only represented a small number of products overall.

**Table 2:** Prevalence of sustainability claims overall and by product category

Product category	Number of products^*^	Number of products displaying ≥ 1 claim	Proportion of products displaying ≥ 1 claim (%)^†^	Total number of different claims identified^‡^	Variety of claims^§^	Three most common claims identified
All product categories	27 738	10 781	39	17 423	69	Natural Vegan Organic
Bread and bakery products	2889	904	31	1270	30	Vegan Natural Sustainable palm oil
Cereal and grain products	1965	788	40	1213	27	Vegan Natural Organic
Confectionery	1698	849	50	1387	30	Rainforest Alliance Certified Natural Cocoa Life Certified
Convenience foods	1857	477	26	697	29	Vegan Natural Sustainable
Dairy	3173	1133	36	1814	34	Natural Vegan Organic
Edible oils and oil emulsions	461	194	42	347	19	Natural Vegan Organic
Egg and egg products	84	75	89	118	9	Free range Natural Cage free
Foods for specific dietary use	855	440	51	844	27	Natural Organic Vegan
Fruits, vegetables, nuts and legumes	4218	1548	37	2530	33	Natural Vegan Organic
Meat and meat alternatives	1983	689	35	1268	30	Free range Vegan Natural
Meat	1722	472	27	748	19	Free range Natural RSPCA (Royal Society for the Prevention of Cruelty to Animals) Approved
Meat alternatives	261	217	83	520	26	Vegan Plant based GMO free
Non-alcoholic beverages	2981	1343	45	2151	34	Natural Vegan Organic
Sauces, dressings, spreads and dips	2805	1075	38	1748	28	Vegan Natural Organic
Seafood and seafood products	927	365	39	494	23	Marine Stewardship Council Certified Natural Sustainable
Snack foods	1322	641	48	1082	27	Vegan Natural Plant based
Sugars, honey and related products	520	260	50	460	20	Natural Vegan Organic

* Number of all products in product category (both labelled and unlabelled).

† Number of products displaying at least one claim as a proportion of all products in product category.

‡ Number of different claims on each product package, totalled across each product category and the overall sample. Repeated claims (e.g. the claim ‘vegan’ stated on the product name and elsewhere on pack) were counted once. A product displaying ‘carbon friendly’, ‘natural’ and ‘vegan’ was considered to have three different claims on pack.

§ Number of claims identified at least once, overall and within a product category. Refer to online supplementary material, Supplemental Table 3 for full list of claims by product category and the number of products displaying each claim.

By major food category, the highest number of claims identified was in the fruit, vegetables, nuts and legumes category (*n* 2530, see Table 2). Categories with the largest proportion of products carrying sustainability claims were egg products (89 %) and foods for specific dietary use (e.g. protein powders and protein bars, 51 %). ‘Free-range’ claims were the most common sustainability claim on eggs, while ‘natural’ claims were the most frequently identified sustainability claim on foods for specific dietary use (see Table 2). By sub-category, meat alternatives had a high prevalence of claims (83 % of products displaying at least one claim), with the ‘vegan’ and ‘plant-based’ claims the most prevalent (see Table 2). Only 27 % of meat products displayed at least one claim, the most prevalent claims being ‘free-range’ and ‘natural’ (see Table 2). A wide variety of claims was observed in most product categories. The dairy product category and non-alcoholic beverages category had the largest variety of claims (both categories had a variety of thirty-four claims). By contrast, the egg products, edible oils and oil emulsions and meat categories had the lowest variety of claims (variety of nine, nineteen and nineteen claims, respectively).

### Prevalence by sustainability domains and type

#### Sustainability domains

Among the total number of sustainability claims identified, 59 % were multi-domain (*n* 10 316, see Table 3). The most prevalent claims within the multi-domain category were ‘vegan’ claims, followed by ‘organic’ claims (36 % and 17 % of claims in this domain respectively, see Table 3).

**Table 3:** Prevalence of sustainability claims by sustainability domain

Sustainability domain	Total number of different claims identified^*^	Proportion of all identified claims (%)^†^	Variety of claims^‡^	Three most common claims identified in each domain (%)^§^
All sustainability domains	17 423	100	69	Natural (24 %) Vegan (21 %) Organic (10 %)
Multi-domain	10 316	59	22	Vegan (36 %) Organic (17 %) Australian Certified Organic (10 %)
Environment	4859	28	29	Natural (85 %) Marine Stewardship Council Certified (4 %) Carbon Neutral Certified (2 %)
Generic	1513	9	10	Sustainable (35 %) Sustainable palm oil (26 %) Free from palm oil (22 %)
Animal welfare	636	4	7	Free range (54 %) RSPCA (Royal Society for the Prevention of Cruelty to Animals) Approved (28 %) No antibiotics (9 %)
Social	99	1	1	Fairtrade (100 %)

* Number of different claims on each product package, totalled across each product category and the overall sample. Repeated claims (e.g. the claim ‘vegan’ stated on the product name and elsewhere on pack) were counted once. A product displaying ‘carbon friendly’, ‘natural’ and ‘vegan’ was considered to have three different claims on pack.

† Percentage distribution of the frequency of different claims.

‡ Number of different claims identified at least once, overall and by sustainability domain. Refer to online supplementary material, Supplemental Table 2 for full list of claims and their allocated sustainability domain.

§ Proportion of the total number of different claims identified in each sustainability domain.

The environmental domain was the second most prevalent domain category (28 %) but had the largest variety of claims (*n* 29). The most common claim within the environment domain was ‘natural’ both overall (85 % of claims in this domain, see Table 3) and across six of fifteen product categories (see Table 2, and online supplementary material, Supplemental Table 3 for the claims by product category and the number of products displaying each claim). The sugars and honey product category had the highest proportion of environment domain claims (30 % of products in this category, mostly ‘natural’ claims, data not shown).

The generic sustainability domain represented 9 % of all claims. The most common claim within this domain was the ‘sustainable’ claim (35 % of claims in this domain).

The animal welfare domain comprised 4 % of all claims identified. The most prevalent claim in this domain was ‘free range’ (54 %). The majority of claims in this domain were derived from two product categories, meat and meat alternatives and egg products. There was low prevalence of claims referring to the social domain (1 %), largely due to these claims incorporating aspects relevant to other domains and hence being categorised as multi-domain.

#### Types of sustainability claims

**Figure 1:**
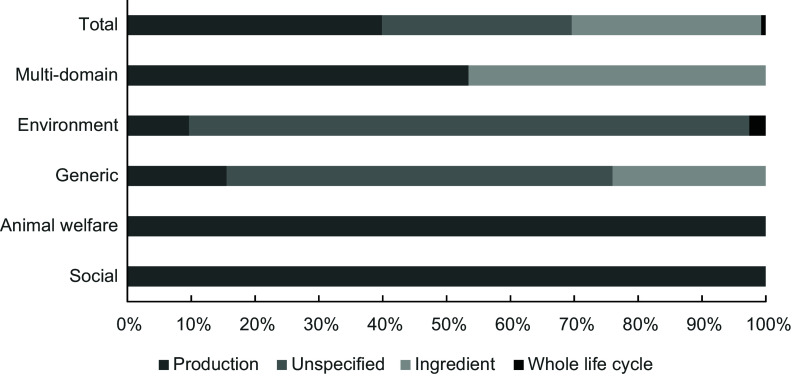
Proportions of sustainability claims identified overall and by sustainability domain and type of claim.

Despite the prevalence of claims being generally evenly distributed across production, ingredient and unspecified claim types (see Figure 1), there was considerable variation in the breadth of claims, from eight to forty-five claims (see online supplementary material, Supplemental Table 4). Claims referring to the production stages were the most common (40 % of all claims identified, see online supplementary material, Supplemental Table 4). A wide variety of claims referred to production stages (*n* 45); however, many of these claims had low prevalence (nineteen claims featured on ≤ 10 products, see online supplementary material, Supplemental Table 2). Unspecified claims accounted for 30 % of claims and were mostly ‘natural’ claims (85 % of unspecified claims). Despite the prevalence of ingredients claims (30 % of claims), there were only eleven unique claims relating to ingredients, with most claims in this category referring to veganism or the absence of meat products (e.g. ‘plant based’, ‘no animal content’). Whole life cycle claims were observed only in the environmental domain, as they all referred to life cycle assessments for GHGe (< 1 % of all claims identified).

### Text and logo formats

Text claims were much more frequently observed than logos. In total, 14 690 text claims were displayed (84 % of all different claims identified, see Table 4). There was a wide variety of sustainability claims identified, consisting of forty-nine text and twenty logo claims. The most prevalent text claims were ‘natural’ (28 % of text claims identified) and ‘vegan’ claims (25 %). By product category, the egg category had the highest proportion of text claims (77 % of products, see online supplementary material, Supplemental Table 5).

**Table 4:** Prevalence of sustainability claims by format

Format	Total number of different claims identified^*^	Proportion of all identified claims (%)^†^	Variety of claims^‡^	Three most common claims identified for each format (%)^§^
Total	17 423	100	69	
Text	14 690	84	49	Natural (28 %) Vegan (25 %) Organic (12 %)
Logo	2733	16	20	Australian Certified Organic (38 %) Rainforest Alliance Certified (27 %) Marine Stewardship Council Certified (7 %)

* Number of different claims on each product package, totalled across each product category and the overall sample. Repeated claims (e.g. the claim ‘vegan’ stated on the product name and elsewhere on pack) were counted once. A product displaying ‘carbon friendly’, ‘natural’ and ‘vegan’ was considered to have three different claims on pack.

† Percentage distribution of the frequency of different claims.

‡ Number of claims identified at least once, overall and by format. Refer to online supplementary material, Supplemental Table 2 for full list of claims and their allocated format.

§ Proportion of the total number of different claims identified in each format.

In total, 2733 logos were displayed. Most logos were third-party certifications, with the exception of in-house logo claims observed on chocolate and confectionery products (6 % of all logo claims, data not shown). The most prevalent logo was ‘Australian Certified Organic’ (38 % of all logo claims). While text claims were generally prevalent across most product categories (see Table 2), the ‘Rainforest Alliance Certified’ logo was the most prevalent claim in the confectionery product category (13 % of products, data not shown) and the ‘Marine Stewardship Council Certified’ claim was the most prevalent claim in the seafood product category (20 % of products, data not shown).

## Discussion

This study found widespread use of sustainability claims on Australian packaged foods, with approximately one-third of products displaying at least one sustainability claim. Two text claims alone, ‘vegan’ and ‘natural’, comprised nearly 50 % of the total number of different claims identified, although there was a wide variety of claims observed overall. The most prevalent claims applied to more than one sustainability domain, most often environmental and animal welfare concerns, such as ‘vegan’ claims.

The vagueness of the term ‘natural’ may explain why it featured frequently across all product categories. Use of this claim is particularly problematic because it is unknown what part of the product or its production process is ‘natural’. The ‘vegan’ claim was prevalent on a large number of inherently vegan products (i.e. fruits, vegetables, nuts and legumes). Use of the ‘vegan’ claim is unsurprising given the increasing number of consumers seeking out vegan products for sustainability and health benefits^(28)^. However, both the ‘natural’ and ‘vegan’ claims provide limited insight into production processes or broader sustainability impact, which require more rigorous oversight and verification.

### Risk of consumer confusion

Failing to transition to a sustainable food system will obstruct the attainment of the Paris Agreement target to limit global warming to 2°C^(3)^. Meaningful and reliable information on food sustainability is crucial to facilitate consumers’ transition to healthy and sustainable dietary patterns^(9,11)^. Existing guidance posits that sustainability labels should be substantiated with evidence, facilitate meaningful comparisons and incorporate a product’s life cycle impact, rather than taking a piecemeal approach biased towards positive attributes^(7,10,29)^.

It is evident from our analysis that many claims identified are inherently challenging to substantiate. The common use of vague, unspecified claims such as ‘natural’ and generic terms such as ‘sustainable’ is concerning, with lack of an agreed definition of those terms making them difficult to substantiate. Further, claims relating to production processes and life cycle approaches cannot be easily substantiated unless endorsed by an independent third party. Despite the role of independent verification to substantiate claims and improve perceived credibility, logos accounted for only 16 % of total claims identified in our sample. Claims relating to product ingredients could be cross-checked against ingredient listings on-pack, such as the ‘vegan’ claim. However, ingredients need to be provided accurately and understandably for consumers in order to be meaningful. Over two-thirds of claims were related to production processes or lacked specificity and hence cannot be easily verified. Altogether, the challenges identified in substantiating claims supports concerns raised in Australia by the ACCC around broad and unqualified claims like ‘eco-friendly’ and ‘sustainable’^(18)^. The results are also consistent with work by the European Commission that found that 53 % of sustainability claims displayed on consumer goods in Europe were vague, misleading or unverified^(30)^.

We found a wide variety of claims, including within product category, likely hindering the ability of consumers to compare the sustainability of different products. For example, within the dairy product category alone, the sustainability claims identified included ‘natural’, references to palm oil and generic use of the term ‘sustainability’, in addition to text and logo formats of both the ‘vegan’ and ‘organic’ claims. This vast array of claims can lead to consumer confusion. The low prevalence of claims incorporating a whole life cycle approach indicates the complexities of calculating comprehensive life-cycle impacts, suggesting research is needed in this area^(6)^.

Our findings that the majority of existing sustainability claims are unverified and vague add important context to research examining the influence of claims on consumer behaviour. Previous research highlights that consumer use of labels is influenced by the perceived trustworthiness of the endorsing entity^(21,31)^. This suggests that the unverified sustainability claims currently displayed may not be trusted. The wide variety of broad claims is also concerning given existing work showing that consumers are prone to misunderstanding the nuances of different labels^(32)^. Taken together, these shortcomings highlight policy gaps in sustainability labelling, thereby increasing the risk of ‘greenwashing’. This is problematic, given that misleading and ambiguous claims erode consumer trust and weaken consumers’ ability to make informed choices^(10,21)^. For food manufacturers, greenwashing also creates unfair competition for producers who adopt genuinely more sustainable practices.

### Recommendations for policymakers

One mechanism to improve the utility of sustainability labels to consumers is through stronger regulation. Lessons here can be drawn from health and nutrition labelling of food, where similar concerns around the need to ensure labelling was truthful and not misleading led to the development of international food standards^(15,16)^. Codex standards now offer guidance on informational nutrient declarations, the regulation of health and nutrition claims and the development of front-of-pack nutrition labels^(15,16)^. These standards form the basis of legislation adopted by many countries at the national level^(33,34)^. By contrast, similar international norms are not yet available for policymakers interested in progressing regulation of sustainability labels.

Improved regulation in a sustainability context could include more stringent criteria for foods to meet before being eligible to display specific text claims. In Australia, regulatory guidance exists for some terms such as ‘free range eggs’ under the Australian Consumer Law, or exports labelled as ‘organic’^(35,36)^. However, most claims (including common ones such as ‘vegan’ and ‘natural’) lack centrally legislated definitions or criteria for use, making it difficult for consumers to interpret what these claims mean.

Other regions have implemented legislated definitions for some sustainability claims. For example, in the European Union and United Kingdom, displaying an ‘organic’ claim requires certain production criteria to be met^(37,38)^. In the USA, the Department of Agriculture defines common terms such as ‘natural’ for meat and poultry products^(39)^. In a health and nutrition context, there are regulated criteria, for example, for amounts of specific nutrients in a product to make a nutrition claim (e.g. ‘high in protein’), and details are provided so jurisdictions can develop and implement evidence-based processes to substantiate health claims^(15)^. Similar government-backed consensus for frequently used sustainability terms could help guide both consumers and manufacturers. For consumers, it can reduce confusion, and for manufacturers, definitions would provide guidance on when these terms are permitted, levelling the playing field and reducing incentives to greenwash to obtain a competitive advantage.

Another policy option is to implement a holistic government-led sustainability label, similar to regulatory innovation in front-of-pack nutrition labelling worldwide. At least thirty-five countries have adopted a government-endorsed front-of-pack nutrition label that uses interpretive information such as colours and symbols to provide at-a-glance nutrition information^(40)^. For example, Australia’s existing front-of-pack nutrition label, the Health Star Rating, summarises a product’s healthiness from 0·5 stars (least healthy) to 5·0 stars (most healthy) using an overall algorithm. Other countries, including many in the Americas, have implemented ‘stop-sign’ warning labels on foods that contain excessive amounts of risk nutrients, and the use of colour has been found to be effective in distinguishing unhealthy and healthier products^(40,41)^. A similar example of a sustainability label that summarises overall impacts is the French Eco-Score^(12)^. These types of labels that provide objective information, including on less favourable elements of a product (i.e. unhealthiness or unsustainability), can be distinguished from the sustainability claims in the current study that highlight favourable elements only^(40,42)^. However, in order to be effective as a policy tool, government-led labels should be mandatory^(43,44)^. Through mandating a label, less sustainable products are also compelled to display low scores so consumers can compare products and make informed decisions. A mandatory front-of-pack sustainability label may be preferable to the current wide variety of voluntary claims in providing consumers meaningful information^(45)^.

While much can be learned from experiences with health and nutrition labelling, there are additional challenges that must be recognised in the sustainability context. Given the inherent breadth and complexity of sustainable food systems, any policy initiative requires agreement on the definition of sustainability and identifying the relevant government department responsible for sustainability labelling. Common claims such as ‘natural’ and ‘vegan’ have overlapping health and sustainability attributes, thereby blurring the appropriate regulating body for such claims. This regulatory gap may explain why both ‘natural’ and ‘vegan’ are not defined by either the Food Standards Australia New Zealand, the food standards body in Australia or under Australian Consumer Law. Considering the potential for multiple departments to be involved, it is inevitable that food systems trade-offs must be navigated. Regardless of these caveats, there is acknowledgement globally for the need for improved guidance on the use of sustainability labels^(7,46)^. Codex has initiated investigations into sustainability labelling^(7)^, and the European Commission is looking to harmonise the use of labels^(46)^. Overall, the nutrition labelling context provides potential pathways for a government-led sustainability label in Australia, providing that the system is evidence based.

### Strengths and limitations

A key strength of this study is the large number of products collected from major food retailers, collectively representing over 80 % market share in Australia^(47)^. The FoodSwitch program’s systematic data collection by trained data collectors enabled a large number of claims to be captured, allowing comprehensive analysis of the prevalence of sustainability claims. Another study strength is utilising the internationally developed INFORMAS labelling taxonomy to categorise sustainability labels. Leveraging a standardised protocol permits global comparison with other regions and provides a basis for monitoring the types of sustainability labels identified.

Limitations include the scope of claims, which was restricted to the sustainability claims captured in the FoodSwitch dataset. Claims were collected prior to development of the INFORMAS categorisation used in this study. Our analysis of prevalence likely underestimates the number of sustainability claims viewed by consumers in practice, given that claims were counted only once per package where in fact they may be displayed on a product’s packaging multiple times. Future research can include claims that did not refer to the product, and hence were beyond the scope of this study (such as packaging claims). Accurately categorising each claim by sustainability domain and type required a pragmatic approach. This was particularly the case for self-declared text claims without supporting sources on what the claims mean. Online supplementary material, Supplemental Table 1 contains the rationale for categorising each claim and any assumptions made within an Australian context, noting that claims may be categorised differently in other regions (e.g. ‘local’ may be included as a sustainability label in some jurisdictions). The concept and definition of sustainability is also evolving, so the determination of whether a label is sustainability-related may be subject to change.

While consumer behaviour was beyond the scope of the present study, our findings offer opportunities to test the performance of existing sustainability claims against potential alternatives (e.g. interpretive graded labels) on consumer use and understanding. Finally, it is critical that future research evaluates the actual sustainability of products displaying sustainability claims and examines whether differences exist between product categories and label types.

### Conclusion

The prevalence of sustainability claims on food packaging that are generic and broad provides limited utility to consumers. Stronger regulation is needed to ensure that sustainability labels help consumers to make more sustainable choices. Overall, our findings highlight the need for enhanced government oversight to ensure food labels provide meaningful disclosure of a product’s sustainability impact, instead of serving as marketing tools biased towards selective positive attributes. Supporting these sustainable dietary shifts is crucial for achieving Paris Agreement goals, limiting the environmental impact of food systems and improving animal welfare and the social conditions of those involved in the food system.

## Supporting information

10.1017/S1368980026102511.sm001 Keaney et al. supplementary material Keaney et al. supplementary material

## Data Availability

The data for this study are available from FoodSwitch, but restrictions apply to the availability of these data, which were used under licence for the current study and so are not publicly available.

## Supplementary material

For supplementary material accompanying this paper visit https://doi.org/10.1017/S1368980026102511
