# Supplementary material for: Prevalence of sustainability claims on packaged food
Source: Public Health Nutr. 2026 Apr 28;29(1):e113. doi: 10.1017/S1368980026102511 (PMC13434480; doi:10.1017/S1368980026102511)
Supplement: Supplementary file 1 [file S1368980026102511sup001.pdf]

Supplementary Material

Supplementary Table 1

Classification of sustainability domain and type for each claim.

Claim	Sustainability domain	Claim type	Rationale
**Animal welfare**	Animal welfare	Production	Practices (activities) in raising of animals.
**Animal Welfare Certified**	Animal welfare	Production	Practices (activities) in raising of animals.
**Cage free**	Animal welfare	Production	Living conditions during raising of animals (raised without cages).
**Free range**	Animal welfare	Production	Living conditions during raising of animals.
**No antibiotics**	Animal welfare	Production	No antibiotics administered in the production process (activity).
**No hormones or growth promotants**	Animal welfare	Production	No hormones administered in the production process (activity).
**RSPCA (Royal Society for the Prevention of Cruelty to Animals) Approved**	Animal welfare	Production	Farming activities to promote animal welfare.
**Carbon friendly**	Environment	Unspecified	Assume refers to greenhouse gas emissions; type of claim cannot be determined.
**Carbon Neutral Certified**	Environment	Whole life cycle	LCA[e] certification.
**Climate Active Carbon Neutral**	Environment	Whole life cycle	LCA[e] certification.

Claim	Sustainability domain	Claim type	Rationale
**ClimeCo Certified Product**	Environment	Whole life cycle	LCA[e] certification.
**Dolphin friendly**	Environment	Production	Activities occurring during farming, relates to biodiversity.
**Dolphin safe**	Environment	Production	Activities occurring during farming, relates to biodiversity.
**Earth**	Environment	Unspecified	Practices with minimal impact on the earth, however, unclear what type of claim is referred to.
**Eco**	Environment	Unspecified	Practices with eco benefits, however, unclear what type of claim is referred to.
**Eco friendly**	Environment	Unspecified	Practices with eco benefits, however, unclear what type of claim is referred to.
**Environmentally friendly**	Environment	Unspecified	Practices with environmental benefits, however, unclear what type of claim is referred to.
**FAD[a] free**	Environment	Production	Production process without the use of FADs (fish aggregating devices).
**Free from chemicals**	Environment	Production	Claims referring to pesticides and chemicals are activities applied during the production process.
**Free from chemicals and pesticides**	Environment	Production	Claims referring to pesticides and chemicals are activities applied during the production process.

Claim	Sustainability domain	Claim type	Rationale
**Free from herbicides and pesticides**	Environment	Production	Claims referring to pesticides and chemicals are activities applied during the production process.
**Green Tick Certified**	Environment	Production	Certification based on activities during the production process.
**Insecticide free**	Environment	Production	Production process of omitting insecticides.
**Marine Stewardship Council Certified**	Environment	Production	Sustainable fishing practices during farming stage.
**Natural**	Environment	Unspecified	Assume this refers to limited use of pesticides (activity to reduce environmental harm) and no GM, similar to organic. Can be considered a health claim (i.e. no artificial or synthetic ingredients included in the product). No regulated definition exists for natural claims (see https://ers.usda.gov/sites/default/files/_laserfiche/publications/106479/EB-35.pdf?v=91042).
**Nature**	Environment	Production	Assume production practices with limited impact on nature (or the environment).
**No chemical fertilisers, pesticides or synthesised chemicals**	Environment	Production	Claims referring to pesticides and chemicals are activities applied during the production process.
**No chemicals**	Environment	Production	Claims referring to pesticides and chemicals are activities applied during the production process.
**No chemicals or pesticides**	Environment	Production	Claims referring to pesticides and chemicals are activities applied during the production process.

Claim	Sustainability domain	Claim type	Rationale
**No herbicides**	Environment	Production	Claims referring to pesticides and chemicals are activities applied during the production process.
**No pesticides**	Environment	Production	Claims referring to pesticides and chemicals are activities applied during the production process.
**NoCO2[c] Certified**	Environment	Whole life cycle	LCA[e] certification.
**Pesticide free**	Environment	Production	Claims referring to pesticides and chemicals are activities applied during the production process.
**The Carbon Trust**	Environment	Whole life cycle	LCA[e] certification.
**Without pesticides**	Environment	Production	Claims referring to pesticides and chemicals are activities applied during the production process.
**Zero pesticides**	Environment	Production	Claims referring to pesticides and chemicals are activities applied during the production process.
**Fairtrade**	Social	Production	Fairtrade activities include worker conditions and remuneration.
**100% palm oil free**	Generic	Ingredient	Unclear if 'palm oil' refers to practices supporting workers and community rights, minimal land use.

Claim	Sustainability domain	Claim type	Rationale
**Free from palm oil**	Generic	Ingredient	Unclear if 'palm oil' refers to practices supporting workers and community rights, minimal land use.
**No palm oil**	Generic	Ingredient	Unclear if 'palm oil' refers to practices supporting workers and community rights, minimal land use.
**Responsibly fished**	Generic	Production	Unclear if term 'responsible' refers to fish (animal welfare), or the ecosystem in which fishing occurs (environment, biodiversity).
**Responsibly sourced**	Generic	Production	Unclear what domain 'responsible' refers to.
**Responsibly sourced cocoa**	Generic	Production	Unclear what domain 'responsible' refers to.
**Sustainable**	Generic	Unspecified	Cannot be determined what type of claim is referred to.
**Sustainable palm oil**	Generic	Unspecified	Unclear if 'palm oil' refers to practices supporting workers and community rights, minimal land use.
**Sustainably sourced**	Generic	Production	Sourcing activities.
**Animal free**	Multi-domain	Ingredient	Absence of ingredient. Can refer to environmental and animal welfare domains.
**ASC (Aquaculture Stewardship Council)**	Multi-domain	Production	Sustainable fishing practices during farming stage. Practices benefit animal welfare and environment.

Claim	Sustainability domain	Claim type	Rationale
**Australian Certified Organic**	Multi-domain	Production	Organic farming methods (activity). Criteria for certification include animal welfare, no use of GMO's and farming methods that can benefit the environment. Therefore, multi-domain. This claim can also be considered a health claim.<br>Products displaying this certification must comply with national standards in Australia (see https://www.agriculture.gov.au/agriculture-land/farm-food-drought/food/organic-biodynamic).
**B Corp Certification**	Multi-domain	Production	Certification includes social and environmental domains.
**Bonsucro**	Multi-domain	Production	Sustainable production of sugar cane for human rights (social domain), and aims to improve the environmental impact of sugar cane (environmental domain).
**Cocoa Life Certified**	Multi-domain	Production	Although cocoa is an ingredient, categorised as production because the certification program refers to aspirational outcomes that occur in the production stage, such as supporting communities (social domain) and protect landscapes (environmental domain).
**Fairtrade Certified**	Multi-domain	Production	Although Fairtrade (text label) is social, the certification has expanded to include multiple indicators across animal welfare, social and environmental domains.

Claim	Sustainability domain	Claim type	Rationale
**GMO[b] Free**	Multi-domain	Production	Genetic modification is an activity to modify an organism's genes during the production stage.
**Meat free**	Multi-domain	Ingredient	Absence of ingredient. Can refer to environmental and animal welfare domains.
**No animal content**	Multi-domain	Ingredient	Absence of ingredient. Can refer to environmental and animal welfare domains.
**No animal products**	Multi-domain	Ingredient	Absence of ingredient. Can refer to environmental and animal welfare domains.
**No GMO[c]**	Multi-domain	Production	Genetic modification is an activity to modify an organism's genes during the production stage. Similar to 'organic' claim, not using genetic modification has benefits for animal welfare and the environment.
**Non GM[d]**	Multi-domain	Production	Genetic modification is an activity to modify an organism's genes during the production stage. Similar to 'organic' claim, not using genetic modification has benefits for animal welfare and the environment.
**Organic**	Multi-domain	Production	Organic refers to activities throughout the production process that encourage animal welfare and limits environmental impact. This claim can also be considered a health claim. In Australia, although standards exist for using the organic label, they are voluntary (see https://www.accc.gov.au/consumers/advertising-and-promotions/organic-claims).
**Plant based**	Multi-domain	Ingredient	~~Plant proteins s~~Suggests vegan ingredients, therefore applies to the animal welfare and environmental domains.
**Plant protein**	Multi-domain	Ingredient	Plant proteins suggest vegan ingredients, therefore applies to the animal welfare and environmental domains.

Claim	Sustainability domain	Claim type	Rationale
**Rainforest Alliance Certified**	Multi-domain	Production	Certification criteria include production processes around environmental and social sustainability.
**RSPO (Roundtable on Sustainable Palm Oil)**	Multi-domain	Production	Refers to production processes which align to RSPO standards for sustainable social and environmental impacts.
**RTRS (Roundtable on Responsible Soy)**	Multi-domain	Production	Responsible production activities addressing social and environmental issues.
**UTZ Certified**	Multi-domain	Production	Relates to sustainable farming practices for improved working conditions for farmers (social domain) and protecting the environment (environment domain).
**Vegan**	Multi-domain	Ingredient	Vegan ingredients. Can refer to environmental benefits and animal welfare.<br>Text claims containing the term 'vegan' are included here (e.g., 'vegan friendly'). Vegan Australia Certified products are excluded, unless they also display the 'vegan' text claim.<br><br>This claim can also be considered a health claim. No regulated definition exists for this claim (see https://www.fda.gov/media/184810/download?attachment and https://www.food.gov.uk/safety-hygiene/vegan-food-and-allergens).
**Vegan Australia Certified**	Multi-domain	Ingredient	Vegan ingredients. Can refer to environmental benefits and animal welfare.<br>This claim can also be considered a health claim. No regulated definition exists for this claim, however products must comply with the Vegan Australia Certified standard.

[a] FAD: fish aggregating device

[b] CO_2: carbon dioxide

[c] GMO: genetically modified organism

[d] GM: genetically modified

[e] LCA: life cycle assessment

Supplementary Table 2

Classification of sustainability claims by sustainability domain, type of claim and format, and their prevalence.

**Sustainability domain**	**Claim type**	**Claim**	**Format**	**N**[e]	**Proportion of all products (%)**[f]
Multi-domain	Ingredient	Vegan	Text	3,734	13
Multi-domain	Ingredient	Plant based	Text	960	3
Multi-domain	Ingredient	Vegan Australia Certified	Logo	32	<1
Multi-domain	Ingredient	No animal content	Text	27	<1
Multi-domain	Ingredient	Plant protein	Text	21	<1
Multi-domain	Ingredient	Meat free	Text	15	<1
Multi-domain	Ingredient	No animal products	Text	11	<1
Multi-domain	Ingredient	Animal free	Text	3	<1
Multi-domain	Production	Organic	Text	1,794	6
Multi-domain	Production	Australian Certified Organic	Logo	1,034	4
Multi-domain	Production	GMO [a] free	Text	812	3
Multi-domain	Production	Rainforest Alliance Certified	Logo	729	3
Multi-domain	Production	Non GM[b]	Text	712	3
Multi-domain	Production	Cocoa Life Certified	Logo	156	<1
Multi-domain	Production	B Corp Certification	Logo	140	<1
Multi-domain	Production	RSPO (Roundtable on Sustainable Palm Oil)	Logo	56	<1
Multi-domain	Production	Fairtrade Certified	Logo	41	<1
Multi-domain	Production	UTZ Certified	Logo	25	<1
Multi-domain	Production	Bonsucro	Logo	11	<1

Sustainability domain	Claim type	Claim	Format	N[e]	Proportion of all products (%)[f]
Multi-domain	Production	ASC (Aquaculture Stewardship Council)	Logo	1	<1
Multi-domain	Production	No GMO[a]	Text	1	<1
Multi-domain	Production	RTRS (Roundtable on Responsible Soy)	Logo	1	<1
Environment	Production	Marine Stewardship Council Certified	Logo	198	<1
Environment	Production	Nature	Text	94	<1
Environment	Production	No chemicals	Text	60	<1
Environment	Production	No pesticides	Text	34	<1
Environment	Production	FAD[c] free	Text	18	<1
Environment	Production	Free from chemicals	Text	14	<1
Environment	Production	No chemicals or pesticides	Text	14	<1
Environment	Production	Free from chemicals and pesticides	Text	8	<1
Environment	Production	Pesticide free	Text	7	<1
Environment	Production	Dolphin friendly	Text	6	<1
Environment	Production	Free from herbicides and pesticides	Text	3	<1
Environment	Production	Green Tick Certified	Logo	3	<1
Environment	Production	No chemical fertilisers, pesticides or synthesised chemicals	Text	3	<1
Environment	Production	No herbicides	Text	2	<1
Environment	Production	Without pesticides	Text	2	<1
Environment	Production	Dolphin safe	Text	1	<1
Environment	Production	Insecticide free	Text	1	<1
Environment	Production	Zero pesticides	Text	1	<1

Sustainability domain	Claim type	Claim	Format	N[e]	Proportion of all products (%)[f]
Environment	Whole life cycle	Carbon Neutral Certified	Logo	110	<1
Environment	Whole life cycle	ClimeCo Certified Product	Logo	7	<1
Environment	Whole life cycle	Climate Active Carbon Neutral	Logo	6	<1
Environment	Whole life cycle	NOC02[d] Certified	Logo	1	<1
Environment	Whole life cycle	The Carbon Trust	Logo	1	<1
Environment	Unspecified	Natural	Text	4,126	15
Environment	Unspecified	Earth	Text	82	<1
Environment	Unspecified	Environmentally friendly	Text	30	<1
Environment	Unspecified	Eco	Text	12	<1
Environment	Unspecified	Carbon friendly	Text	11	<1
Environment	Unspecified	Eco friendly	Text	4	<1
Generic	Ingredient	Free from palm oil	Text	340	1
Generic	Ingredient	No palm oil	Text	19	<1
Generic	Ingredient	100% palm oil free	Text	4	<1
Generic	Production	Sustainably sourced	Text	220	<1
Generic	Production	Responsibly fished	Text	11	<1
Generic	Production	Responsibly produced	Text	2	<1
Generic	Production	Responsibly sourced	Text	1	<1
Generic	Production	Responsibly sourced cocoa	Text	1	<1
Generic	Unspecified	Sustainable	Text	527	2
Generic	Unspecified	Sustainable palm oil	Text	388	1

Sustainability domain	Claim type	Claim	Format	N[e]	Proportion of all products (%)[f]
Animal welfare	Production	Free range	Text	342	1
Animal welfare	Production	RSPCA (Royal Society for the Prevention of Cruelty to Animals) Approved	Logo	178	<1
Animal welfare	Production	No antibiotics	Text	56	<1
Animal welfare	Production	No hormones or growth promotants	Text	33	<1
Animal welfare	Production	Cage free	Text	17	<1
Animal welfare	Production	Animal welfare	Text	7	<1
Animal welfare	Production	Animal Welfare Certified	Logo	3	<1
Social	Production	Fairtrade	Text	99	<1

[a] GMO: genetically modified organism
[b] GM: genetically modified
[c] FAD: fish aggregating device
[d] CO_2: carbon dioxide
[e] Unique claims refer to claims identified on each pack at least once. Repeated claims (e.g. the claim ‘vegan’ stated on the product name and elsewhere on pack), were counted once. A product displaying ‘carbon friendly’, ‘natural’, and ‘vegan’ was considered to have three unique claims.
[f] Percentages are shown as whole numbers; values < 1% are reported as < 1

Supplementary Table 3

Prevalence of each sustainability claim by product category. Prevalence was determined by the number of products displaying each claim at least once.

A: Text claims

**Claim**	**Bread and bakery products**	**Cereal and grain products**	**Confectionery**	**Convenience foods**	**Dairy**	**Edible oils and oil emulsions**	**Egg and egg products**	**Foods for specific dietary use**	**Fruits, vegetables, nuts and legumes**	**Meat and meat alternatives**	***Meat***	***Meat alternatives***	**Non-alcoholic beverages**	**Sauces, dressings, spreads and dips**	**Seafood and seafood products**	**Snack foods**	**Sugars, honey and related products**
Natural	187	264	190	124	562	99	20	172	688	157	*134*	*23*	680	401	152	278	152
Vegan	383	369	136	138	276	75	2	108	677	165		*165*	397	613	1	290	104
Organic	64	132	66	27	192	45	10	153	461	94	*77*	*17*	270	151	15	56	58
Plant based	67	72	56	57	142	5		65	105	119		*119*	82	89	1	85	15
GMO[a] free	65	105	54	29	70	18	1	63	80	65	*19*	*46*	58	107	1	79	17
Non GM[b]	51	59	34	19	110	22		86	75	46	*9*	*37*	31	75	4	78	22
Sustainable	35	35	75	73	33	18	3	9	19	20	*10*	*10*	78	31	77	16	5
Sustainable palm oil	169	15	22	31	32	11		11	7	12	*10*	*2*	2	11	2	50	13
Free range	8	22	1	23	12		53	2		166	*166*		1	54			
Free from palm oil	31	12	113	8	21	7		17	6	23	*1*	*22*		47		54	1
Sustainably sourced	6	8	119	8	7			4	17	6	*3*	*3*	22	2	15	4	2
Fairtrade	1	1	44	2	13				1				28	7			2
Nature	6	2	1	28	3			6	2	17		*17*	9	1	1	14	4
Earth	4	6	9	19	1	4			6	2		*2*	1			15	15
No chemicals	4				21	2		1	6	18	*16*	*2*	5	1	1		1
No antibiotics				1	2			1	1	46	*44*	*2*		4	1		
No pesticides	1			7		1		16	5				1	2			1
No hormones or growth promotants										33	*32*	*1*					
Environmentally friendly		6	7					2	1				12			1	1
No animal content	1	2		2	3					2		*2*		13		4	
Plant protein	1	4		1				1	3	10		*10*	1				
No palm oil	5	1	2		1			1					1	8			

Claim	**Bread and bakery products**	**Cereal and grain products**	**Confectionery**	**Convenience foods**	**Dairy**	**Edible oils and oil emulsions**	**Egg and egg products**	**Foods for specific dietary use**	**Fruits, vegetables, nuts and legumes**	**Meat and meat alternatives**	***Meat***	***Meat alternatives***	**Non-alcoholic beverages**	**Sauces, dressings, spreads and dips**	**Seafood and seafood products**	**Snack foods**	**Sugars, honey and related products**
FAD[c] free				4										1	13		
Cage free							17										
Meat free				3						12		*12*					
No chemicals or pesticides				3	11												
Free from chemicals	2	1						1	4				6				
Eco			11			1											
No animal products	1	1	1		1			2	1	3		*3*				1	
Carbon friendly					5	2							3			1	
Responsibly fished									1						10		
Free from chemicals and pesticides									4				4				
Pesticide free					1	1			1				4				
Animal welfare										7	*7*						
Dolphin friendly				1											5		
Eco friendly			1	1		1		1									
100% palm oil free			4														
Animal free	1									2		*2*					
Free from herbicides and pesticides	2				1												
No chemical fertilisers, pesticides or synthesised chemicals													3				
No herbicides									1				1				
Without pesticides									1							1	
Responsibily produced																2	
No GMO[a]																1	
Dolphin safe															1		

Claim	Bread and bakery products	Cereal and grain products	Confectionery	Convenience foods	Dairy	Edible oils and oil emulsions	Egg and egg products	Foods for specific dietary use	Fruits, vegetables, nuts and legumes	Meat and meat alternatives	*Meat*	*Meat alternatives*	Non-alcoholic beverages	Sauces, dressings, spreads and dips	Seafood and seafood products	Snack foods	Sugars, honey and related products
Insecticide free																1	
Zero pesticides		1															
Responsibly sourced cocoa					1												
Responsibly produced												*23*			1		
**Total**	**1,095**	**1,118**	**946**	**609**	**1,521**	**312**	**106**	**722**	**2,173**	**1,025**	***528***	***497***	**1,700**	**1,618**	**301**	**1,031**	**413**

B: Logo claims

Claim	Bread and bakery products	Cereal and grain products	Confectionery	Convenience foods	Dairy	Edible oils and oil emulsions	Egg and egg products	Foods for specific dietary use	Fruits, vegetables, nuts and legumes	Meat and meat alternatives	*Meat*	*Meat alternatives*	Non-alcoholic beverages	Sauces, dressings, spreads and dips	Seafood and seafood products	Snack foods	Sugars, honey and related products
Australian Certified Organic	22	74	32	13	127	29	9	85	332	67	*54*	*13*	132	73	1	10	28
Rainforest Alliance Certified	125	4	213		108			20	1				218	6	1	26	7
Marine Stewardship Council Certified				8											187	3	
RSPCA (Royal Society for the Prevention of Cruelty to Animals) Approved	3	1		41				1	1	122	*121*	*1*		9			
Cocoa Life Certified			148		2	1			2				2			1	
B Corp Certification		12	12	21	23			12	16	4	*1*	*3*	19	16		2	3
Carbon Neutral Certified			2	1	16		3			40	*40*		42	3	2	1	
RSPO (Roundtable on Sustainable Palm Oil)	20	3	8	4				3		3	*1*	*2*		7		8	
Fairtrade Certified			19		9								13				
Vegan Australia Certified	2		3		3	5			2	1		*1*	2	14			
UTZ Certified	2		2						1				19	1			
Bonsucro					2												9
ClimeCo Certified Product		1			2			1	2					1			
Climate Active Carbon Neutral			2										3		1		
Green Tick Certified										3		*3*					
Animal Welfare Certified										3	*3*						
The Carbon Trust					1												
ASC (Aquaculture Stewardship Council)															1		

Claim	Bread and bakery products	Cereal and grain products	Confectionery	Convenience foods	Dairy	Edible oils and oil emulsions	Egg and egg products	Foods for specific dietary use	Fruits, vegetables, nuts and legumes	Meat and meat alternatives	*Meat*	*Meat alternatives*	Non-alcoholic beverages	Sauces, dressings, spreads and dips	Seafood and seafood products	Snack foods	Sugars, honey and related products
NoCO2[d] Certified													1				
RTRS (Roundtable on Responsible Soy)	1																
**Total**	**175**	**95**	**441**	**88**	**293**	**35**	**12**	**122**	**357**	**243**	***220***	***23***	**451**	**130**	**193**	**51**	**47**

[a] GMO: genetically modified organism
[b] GM: genetically modified
[c] FAD: fish aggregating device
[d] CO_2: carbon dioxide

Supplementary Table 4

Prevalence of sustainability claims by type of claim.

Claim type	Total number of different claims identified[a]	Proportion of all claims identified (%)[b]	Variety of claims[c]	Proportion of variety of claims (%)[d]
**All types of claims**	**17,423**	**100**	**69**	**100**
Production	6,952	40	45	65
Unspecified	5,180	30	8	12
Ingredient	5,166	30	11	16
Whole life cycle	125	1	5	7

[a] Number of different claims on each product package, totalled across each product category and the overall sample. Repeated claims (e.g. the claim 'vegan' stated on the product name and elsewhere on pack), were counted once. A product displaying 'carbon friendly', 'natural', and 'vegan' was considered to have three different claims on pack.
[b] Percentage distribution of the frequency of different claims.
[c] Number of different claims identified at least once, overall and by claim type. Refer to **Supplementary Table 2** for full list of claims and their allocated claim type.
[d] Percentage distribution of the variety of claims.

Supplementary Table 5

Proportion of products displaying at least one sustainability claim, by format and product category.

Product category	Proportion of products displaying text claims (%)	Proportion of products displaying logo claims (%)	Proportion of products displaying both formats (%)[a]	Proportion of products displaying no claims (%)	All products in category (%)
**All product categories**	30	3	6	61	**100**
Bread and bakery products	25	3	3	69	100
Cereal and grain products	35	1	4	60	100
Confectionery	26	13	12	50	100
Convenience foods	21	2	2	74	100
Dairy	27	4	5	64	100
Edible oils and oil emulsions	34	0	7	58	100
Egg and egg products	77	0	12	11	100
Foods for specific dietary use	38	1	12	49	100
Fruits, vegetables, nuts and legumes	28	0	8	63	100
Meat and meat alternatives	23	6	6	65	100
Meat	*16*	*6*	*5*	*73*	*100*
Meat alternatives	*74*	*1*	*9*	*17*	*100*
Non-alcoholic beverages	31	5	8	55	100
Sauces, dressings, spreads and dips	34	1	4	62	100
Seafood and seafood products	19	13	8	61	100
Snack foods	45	1	3	52	100
Sugars, honey and related products	41	3	6	50	100

[a] Products displaying a combination of both text and logo formats.
